# Video evidence that cuckoos farm their hosts by ejecting nestlings

**DOI:** 10.1002/ece3.11196

**Published:** 2024-04-04

**Authors:** Jinggang Zhang, Peter Santema, Jianqiang Li, Wenhong Deng, Bart Kempenaers

**Affiliations:** ^1^ Ministry of Education Key Laboratory for Biodiversity Sciences and Ecological Engineering, College of Life Sciences Beijing Normal University Beijing China; ^2^ Department of Ornithology Max Planck Institute for Biological Intelligence Seewiesen Germany; ^3^ Department of Zoology, Edward Grey Institute University of Oxford Oxford UK; ^4^ School of Ecology and Nature Conservation Beijing Forestry University Beijing China

**Keywords:** brood parasitism, common cuckoo, Daurian redstart, farming behaviour, host‐nestling ejection

## Abstract

When host nests are scarce, avian brood parasites would benefit from behaviours that increase the availability of suitable nests. Several studies reported ejection of host nestlings from nests by brood parasites; however, whether brood parasites do so to induce the host to re‐nest and thus increase opportunities for future parasitism (i.e. ‘farming’ behaviour) remains unclear. Here, we report observational evidence of farming behaviour by a common cuckoo *Cuculus canorus* female in a Daurian redstart *Phoenicurus auroreus* population: (1) the cuckoo destroyed a host nest by ejecting all nestlings, (2) the host then produced a new nest and (3) the cuckoo successfully parasitized the replacement nest. We suggest that farming behaviour may be more common, but often goes undetected because it requires intense nest monitoring.

## Graphical abstract

The farming hypothesis suggests that avain brood parasites may destroy nests that are unsuitable for parasitism, thereby inducing the host to re‐nest. In this study, we provided the first explicit evidence that common cuckoos farm Daurian redstarts by ejecting host nestlings.

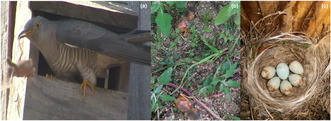

## 1 INTRODUCTION

Avian brood parasites lay their egg in the nest of other species and thus transfer the costs of parental care to their hosts. This selects for defensive host adaptations which, in turn, leads to counteradaptations in the parasitic species (Davies, 2000). These reciprocal interactions between brood parasites and their hosts form a classic model of coevolution (Soler, 2014). When opportunities for parasitism are limited, behaviours that increase the availability of suitable host nests should be selected for in brood parasites. Although brood parasites are typically much less numerous than their hosts, suitable host nests are likely to be scarce before and after the peak egg‐laying period when nesting among hosts is synchronised. The ‘farming hypothesis’ suggests that brood parasites may benefit from destroying host nests that are unsuitable for parasitism (i.e. nests in late incubation or with nestlings) (Arcese et al., 1996; Zahavi, 1979). By ejecting the eggs and/or nestlings, the parasite can induce the host to re‐nest and thereby increases the opportunity for future parasitism (Hauber, 2014; Soler et al., 2017).

However, evidence of farming behaviour is generally scarce. Clear evidence has only been found in the brown‐headed cowbird *Molothrus ater* and only at the egg stage (Hoover & Robinson, 2007; Swan et al., 2015). For instance, a study that presented captive cowbirds with artificial nests with either freshly laid or highly developed host eggs (i.e. simulating suitable and unsuitable nests for parasitism), found that cowbirds punctured or removed the developed eggs more often (Swan et al., 2015). Another study reported that re‐nesting attempts from hosts whose previous nest had been destroyed by a cowbird were parasitized more frequently than other nesting attempts (Hoover & Robinson, 2007). Farming behaviour may also occur in other parasitic species and during the nestling stage of the host, especially because it does not require the parasite's ability to estimate the developmental stage of host eggs (Swan et al., 2015). However, hitherto, there is no direct evidence for farming behaviour at the nestling stage.

Several studies have reported the removal of host nestlings from the nest by adult brood parasites (including in the two most frequently studied species, the common cuckoo *Cuculus canorus* and the brown‐headed cowbird; Šulc et al., 2020). Although this behaviour has sometimes been interpreted as ‘farming’, alternative explanations cannot be excluded (Šulc et al., 2020). For instance, the destruction of the host's breeding attempt may represent a form of ‘punishment’ by the brood parasite of a host that ejected a parasitic egg (the ‘mafia hypothesis’; Soler et al., 1995; Zahavi, 1979). Alternatively, the nestlings might have been eaten by the brood parasite, and the behaviour may thus represent a form of ‘foraging’ for nutrients (Wyllie, 1975; but see Šulc et al., 2020).

To qualify as farming behaviour three lines of evidence are necessary: (1) evidence that the brood parasite destroys a host nest by ejecting the nestlings, (2) evidence that the host produces a replacement nest following the destruction of its previous nest and (3) evidence that the brood parasite parasitizes the re‐nesting attempt. Here, we report on a case of farming behaviour in the common cuckoo with a Daurian redstart *Phoenicurus auroreus* host in which all three conditions are met.

Daurian redstarts are a regular host of common cuckoos (hereafter ‘cuckoos’) in north‐eastern China. They typically produce two clutches within a single breeding season, with the first peak of egg laying in late April and the second peak in early June (Zhang, Møller, et al., 2021; Zhang, Santema, et al., 2021). Common cuckoos generally arrive at the breeding grounds around mid‐May, when most redstart nests are either in late incubation or at the nestling stage of their first clutch (Zhang, Møller, et al., 2021; Zhang, Santema, et al., 2021). The parasitism rate, therefore, varies from no parasitism occurring in the first egg‐laying period, to 15.6% of nests being parasitized during the second laying period (Zhang, Møller, et al., 2021; Zhang, Santema, et al., 2021). It has been shown that cuckoos can farm Daurian redstarts by removing host eggs (Zhong et al., 2019), but the farming behaviour in nestling stage has never been reported in this system.

## 2 METHODS

The population of Daurian redstarts that we studied was in ShuangYu, a village in north‐eastern China (43°37′19″ N and 126°09′54″ E). The study site is about 300 ha and consists of 8 subplots of inhabited land surrounded by arable fields and secondary forest. Daurian redstarts breed at high density near or in the village. They generally build nests in concealed sites, including cavities in trees, rocks, walls and eaves, but they also readily accept artificial nest‐boxes (Zhang, Møller, et al., 2021; Zhang, Santema, et al., 2021). From 2016 to 2021, we placed 200 nest‐boxes in the study site, with a distance between adjacent boxes of about 50 m. Since 2018, we monitored the reproductive behaviour of redstarts throughout the entire breeding season. During the egg‐laying and early incubation stage in the second laying period, we checked the nests at least once a day to assess the parasitism status. As part of the ongoing project, we caught adults 4 days after hatching and banded them with a metal ring and a unique combination of colour rings (Zhang, Møller, et al., 2021; Zhang, Santema, et al., 2021). During the second egg‐laying period from 2018 to 2021, we filmed nests (*N* = 247) when the nestlings were 5 days old as part of a study on parents' provisioning behaviour. To this end, we placed a video camera (SAMSUNG, SMX‐F50) about 3 m in front of the nest and recorded the nest for 2 h during the morning (between 08:00 and 11:00 GMT +8).

## 3 RESULTS

**FIGURE 1:**
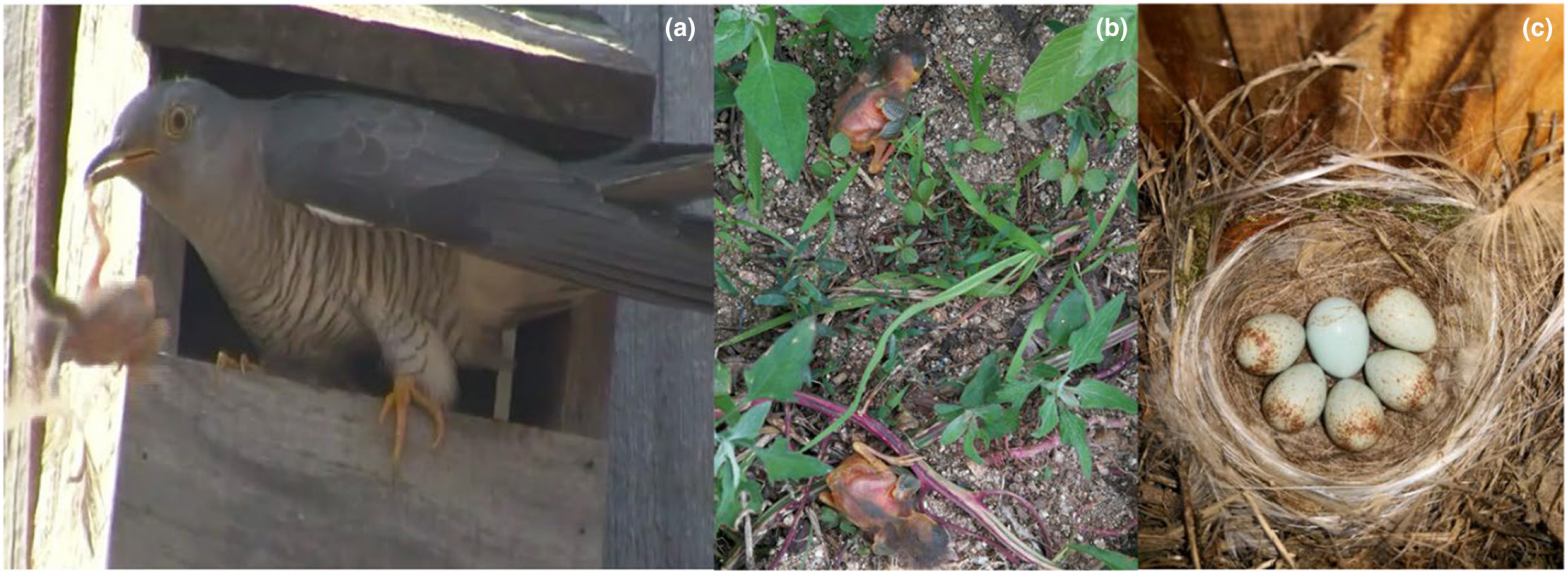
(a) The female common cuckoo ejecting a Daurian redstart nestling from the nest (picture extracted from Video 1); (b) two of the ejected redstart nestlings found on the ground under the nest; (c) a common cuckoo egg in the replacement nest of the same pair (central egg, surrounded by five redstart eggs; one host egg had been removed by the cuckoo during parasitism).

A pair of Daurian redstarts (female B2205261 and male B2205262) had a nest in our study site with seven nestlings that had hatched on 12 June, 2021. When the nestlings were 5 days old (17 June), we recorded an adult common cuckoo that visited the nest and ejected all host nestlings (Video 1). The video shows that the cuckoo ejected the nestlings one by one within a period of less than 1 min. The focal redstart parents emitted intense alarm calls, but they did not show any physical attacks towards the cuckoo. Two days after the nestlings had been ejected (19 June), we found a new nest under construction by the same pair at a distance of approximately 5 m from the old nest. On 20 June, the female laid the first egg in this new nest. Five days after the onset of laying, that is on 25 June when the replacement nest contained 6 eggs, we found a cuckoo egg in the nest (one host egg was removed by the cuckoo; Figure 1c). The redstart female accepted the cuckoo egg, but the nest was later found depredated (1–2 July, i.e. 8–9th day of the incubation stage).

**VIDEO 1:** A female common cuckoo removes all host nestlings from a Daurian redstart nest.

We note that the cuckoo in the video can be identified as a female based on its appearance, that is a brown tinge on the nape, some barring on the upper breast and neck, a blurred demarcation between the barred chest and the grey hood and a paler yellow iris (Figure 1a) (del Hoyo et al., 2020; Noh et al., 2016). Moreover, although we could not confirm with certainty that the cuckoo ejecting the nestlings and laying the egg in the new nest was the same individual, two lines of indirect evidence suggest that it is highly likely. First, our field observations showed that there was only one female cuckoo in this subplot of the study area, as we never observed more than one individual in the subplot at the same time. Second, all cuckoo eggs in the subplot – including the one in the re‐nesting attempt – had the same appearance, suggesting that all the eggs were laid by the same female cuckoo (Šulc et al., 2022).

## 4 DISCUSSION

The suggestion that brood parasites engage in ‘farming’ behaviour to increase opportunities for parasitism within the host population is appealing. The breeding success of cuckoos can be limited by the availability of suitable nests. A cuckoo would then benefit from destroying a nest that is too late in the nesting cycle to be parasitized, if it thereby induces the owners to produce a new nest that it can subsequently parasitize. However, evidence for this behaviour remains scarce. We described a case where (1) a cuckoo destroyed a redstart nest by ejecting the nestlings, (2) the redstart female produced a new nest following destruction of its previous nest and (3) the cuckoo parasitized the re‐nesting attempt. These observations provide rare circumstantial evidence of successful farming behaviour.

The farming event took place in late June, which was about 1 week after the egg‐laying peak of the Daurian redstarts' second breeding attempt, when most redstarts already had nestlings or were in the late incubation stage of their second clutch (Zhang, Møller, et al., 2021; Zhang, Santema, et al., 2021). The timing of this event is thus consistent with the prediction that brood parasites may resort to farming behaviour when the natural availability of nests suitable for brood parasitism is low.

Our observation of a single case of farming behaviour raises the question how prevalent this behaviour is in our population. We observed three additional cases where Daurian redstart nestlings were found on the ground under the nest (as in Figure 1b; one case in 2018 and two cases in 2019). All three cases took place in late June or early July, when the availability of nests suitable for parasitism was low. Although we did not record it, we suspect that nestlings had been ejected by cuckoos, because a predator would have eaten the nestlings. Given that ejected nestlings would presumably quickly disappear (i.e. be consumed by dogs, cats, mice, chickens, …), it is likely that many cases of nest destruction through nestling ejection would have gone undetected. Thus, the prevalence of farming behaviour in our population could be substantially higher than suggested by our ad hoc observations. If so, the host‐nestling ejection by adult cuckoos may not be an accidental behaviour, but may be a more common, adaptive behavioural tactic in this host–parasite system.

Farming behaviour is clearly different from the previously described ‘mafia strategy’ (Soler et al., 1995). Although the latter also includes nest destruction that may induce re‐nesting, the nest destruction is considered a retaliatory behaviour that is only shown if the host does not accept the cuckoo egg. The parasite's ‘mafia’ behaviour can only evolve in parasitic species where the parasitic chick does not kill the host's offspring, such as the great‐spotted cuckoo *Clamator glandarius* (Soler et al., 1995) and the brown‐headed cowbird (Hoover & Robinson, 2007), because only in such systems do the host parents still have the opportunity to successfully raise some of their nestlings after accepting the parasitism (Soler et al., 2017). Thus, the mafia hypothesis cannot explain nest destruction behaviour in the common cuckoo where the cuckoo chick typically evicts all host progeny (Davies, 2000; but see Samaš et al., 2018).

The propensity of farming behaviour may vary among cuckoo populations and depend on traits of the host species. First, farming behaviour may be less likely (or less likely be successful) in host species that aggressively attack the brood parasite. For example, a cuckoo was unable to successfully eject all nestlings from the nest of a wood warbler *Phylloscopus sibilatrix* and from a European robin *Erithacus rubecula*, because of the intense attacks from the host parents (see videos 3 and 4 in Šulc et al., 2020). In contrast, Daurian redstarts do not engage in mobbing or physical attacks against the adult cuckoo (Video 1). Second, farming behaviour might be driven by the mismatch in breeding time between the hosts and the cuckoos. When cuckoos arrive at our study site, Daurian redstarts are normally either in the late incubation or at the nestling stage of their first clutch. One might thus expect an increased incidence of farming behaviour during the late stage of the hosts' first egg‐laying period. However, we did not observe a single (suspected) case during this period. One possible explanation is that the cuckoos were not yet ready for breeding so soon after a long‐distance migration. Third, the frequency of farming behaviour may be linked to the availability of host nests, which may in turn depend on breeding synchrony. While Daurian redstarts occur at high density in our study area, they breed very synchronously (Zhang, Møller, et al., 2021; Zhang, Santema, et al., 2021), such that an abundance of available host nests is quickly followed by a period with few available host nests during which farming behaviour is likely beneficial. As described above, all observed cases of nestling ejection indeed took place after the peak of the redstarts' second egg‐laying period. Last, the frequency of farming behaviour should be related to the probability of re‐nesting. In Daurian redstarts, the laying season extends to late July (or even early August), and parents readily re‐nest when their breeding attempt fails, such that nest destruction by cuckoos likely results in the production of new host nests. Farming behaviour would not be expected in host species that rarely re‐nest.

In summary, we report on one case in which a common cuckoo destroyed a Daurian redstart nest by ejecting all host nestlings, which induced the host to start a new nest, which the cuckoo then successfully parasitized. This suggests that host‐nestling ejection by adult cuckoos can be an effective farming behaviour. We advocate further research in the field using video monitoring or RFID technology to better document and assess the frequency of this behaviour.

## AUTHOR CONTRIBUTIONS

**Jinggang Zhang:** Conceptualization (equal); data curation (equal); funding acquisition (equal); methodology (lead); visualization (lead); writing – original draft (lead); writing – review and editing (lead). **Peter Santema:** Conceptualization (equal); writing – original draft (equal); writing – review and editing (equal). **Jianqiang Li:** Writing – review and editing (equal). **Wenhong Deng:** Conceptualization (equal); funding acquisition (lead); project administration (lead); resources (lead); supervision (equal); writing – original draft (equal); writing – review and editing (equal). **Bart Kempenaers:** Conceptualization (equal); funding acquisition (equal); supervision (equal); writing – original draft (equal); writing – review and editing (equal).

## FUNDING INFORMATION

This study was supported by the National Natural Science Foundation of China (31672297 and 32271559 to W.D.), the China Scholarship Council (201906040159 to J.Z.) and the Max Planck Society (to B.K.).

## CONFLICT OF INTEREST STATEMENT

The authors have no conflict of interest to declare.

## Data Availability

Data sharing is not applicable to this article because no new data was created or analysed in this study.

## References

[ece311196-bib-0001] Arcese, P. , Smith, J. N. , & Hatch, M. I. (1996). Nest predation by cowbirds and its consequences for passerine demography. Proceedings of the National Academy of Sciences of the United States of America, 93, 4608–4611. 11607677 10.1073/pnas.93.10.4608 PMC39325

[ece311196-bib-0002] Davies, N. B. (2000). Cuckoos, cowbirds and other cheats. T. and A.D. Poyser.

[ece311196-bib-0003] del Hoyo, J. , Elliott, A. , Sargatal, J. , Christie, D. A. , & de Juana, E. (2020). Birds of the world. Common Cuckoo (*Cuculus Canorus*), version 1.0. 10.2173/bow.comcuc.01

[ece311196-bib-0004] Hauber, M. E. (2014). Mafia or farmer? Coevolutionary consequences of retaliation and farming as predatory strategies upon host nests by avian brood parasites. Coevolution, 2, 18–25.

[ece311196-bib-0005] Hoover, J. P. , & Robinson, S. K. (2007). Retaliatory mafia behavior by a parasitic cowbird favors host acceptance of parasitic eggs. Proceedings of the National Academy of Sciences of the United States of America, 104, 4479–4483. 17360549 10.1073/pnas.0609710104 PMC1838626

[ece311196-bib-0006] Noh, H. J. , Lee, J. W. , & Yoo, J. C. (2016). Color morph variation in two brood parasites: Common Cuckoo and lesser Cuckoo. Ornithological Science, 15, 109–117.

[ece311196-bib-0007] Samaš, P. , Rutila, J. , Honza, M. , Kysučan, M. , & Grim, T. (2018). Rearing a virulent common cuckoo is not extra costly for its only cavity‐nesting host. Proceedings of the Royal Society B: Biological Sciences, 285, 20181710. 10.1098/rspb.2018.1710 PMC6234889 30355712

[ece311196-bib-0008] Soler, M. (2014). Long‐term coevolution between avian brood parasites and their hosts. Biological Reviews, 89, 688–704. 24330159 10.1111/brv.12075

[ece311196-bib-0009] Soler, M. , Pérez‐Contreras, T. , & Soler, J. (2017). Brood parasites as predators: Farming and mafia strategies. In M. Soler (Ed.), Avian brood parasitism: Behaviour, ecology, evolution and coevolution (pp. 271–286). Springer.

[ece311196-bib-0010] Soler, M. , Soler, J. J. , Martinez, J. G. , & Møller, A. P. (1995). Magpie host manipulation by great spotted cuckoos – evidence for an avian mafia. Evolution, 49, 770–775. 28565143 10.1111/j.1558-5646.1995.tb02312.x

[ece311196-bib-0011] Šulc, M. , Hughes, A. E. , Troscianko, J. , Štětková, G. , Procházka, P. , Požgayová, M. , Piálek, L. , Piálková, R. , Brlík, V. , & Honza, M. (2022). Automatic identification of bird females using egg phenotype. Zoological Journal of the Linnean Society, 195, 33–44.

[ece311196-bib-0012] Šulc, M. , Štětková, G. , Jelínek, V. , Czyż, B. , Dyrcz, A. , Karpińska, O. , Kamionka‐Kanclerska, K. , Rowiński, P. , Maziarz, M. , Gruszczyński, A. , Hughes, A. E. , & Honza, M. (2020). Killing behaviour of adult brood parasites. Behaviour, 157, 1099–1111.

[ece311196-bib-0013] Swan, D. C. , Zanette, L. Y. , & Clinchy, M. (2015). Brood parasites manipulate their hosts: Experimental evidence for the farming hypothesis. Animal Behaviour, 105, 29–35.

[ece311196-bib-0014] Wyllie, I. (1975). Study of cuckoos and reed warblers. British Birds, 68, 369–378.

[ece311196-bib-0015] Zahavi, A. (1979). Parasitism and nest predation in parasitic cuckoos. The American Naturalist, 113, 157–159.

[ece311196-bib-0016] Zhang, J. , Møller, A. P. , Yan, D. , Li, J. , & Deng, W. (2021). Egg rejection changes with seasonal variation in risk of cuckoo parasitism in Daurian redstarts, *Phoenicurus auroreus* . Animal Behaviour, 175, 193–200.

[ece311196-bib-0017] Zhang, J. , Santema, P. , Li, J. , Yang, L. , Deng, W. , & Kempenaers, B. (2021). Host personality predicts cuckoo egg rejection in Daurian redstarts *Phoenicurus auroreus* . Proceedings of the Royal Society B: Biological Sciences, 288, 20210228. 10.1098/rspb.2021.0228 PMC8206684 34130501

[ece311196-bib-0018] Zhong, G. , Wan, G. , Wang, L. , & Liang, W. (2019). Farming behavior by the common cuckoos in its Daurian redstart hosts. Chinese Journal of Zoology, 54, 800–805.

